# When communication all changed

**DOI:** 10.7554/eLife.89322

**Published:** 2023-05-18

**Authors:** John J Dennehy

**Affiliations:** 1 Queens College, City University of New York New York United States

**Keywords:** sparks of change, research culture, equity, diversity, inclusion

## Abstract

Between early challenges and lasting opportunities, a deaf virologist reflects on how the pandemic transformed his access to academic spaces.

On Wednesday March 11, 2020, the governor of New York announced that all in-person classes were to be suspended at my university until the end of the semester. As the news spread, my colleagues speculated that the shutdown might last a few weeks or months. To me it felt more significant, not unlike the aftermath of 9/11 when we realized that the world had irrevocably changed. As a virologist, I had some idea about what was to come; as a deaf individual, I did not fully comprehend what this would entail for me.

**Figure 1:**
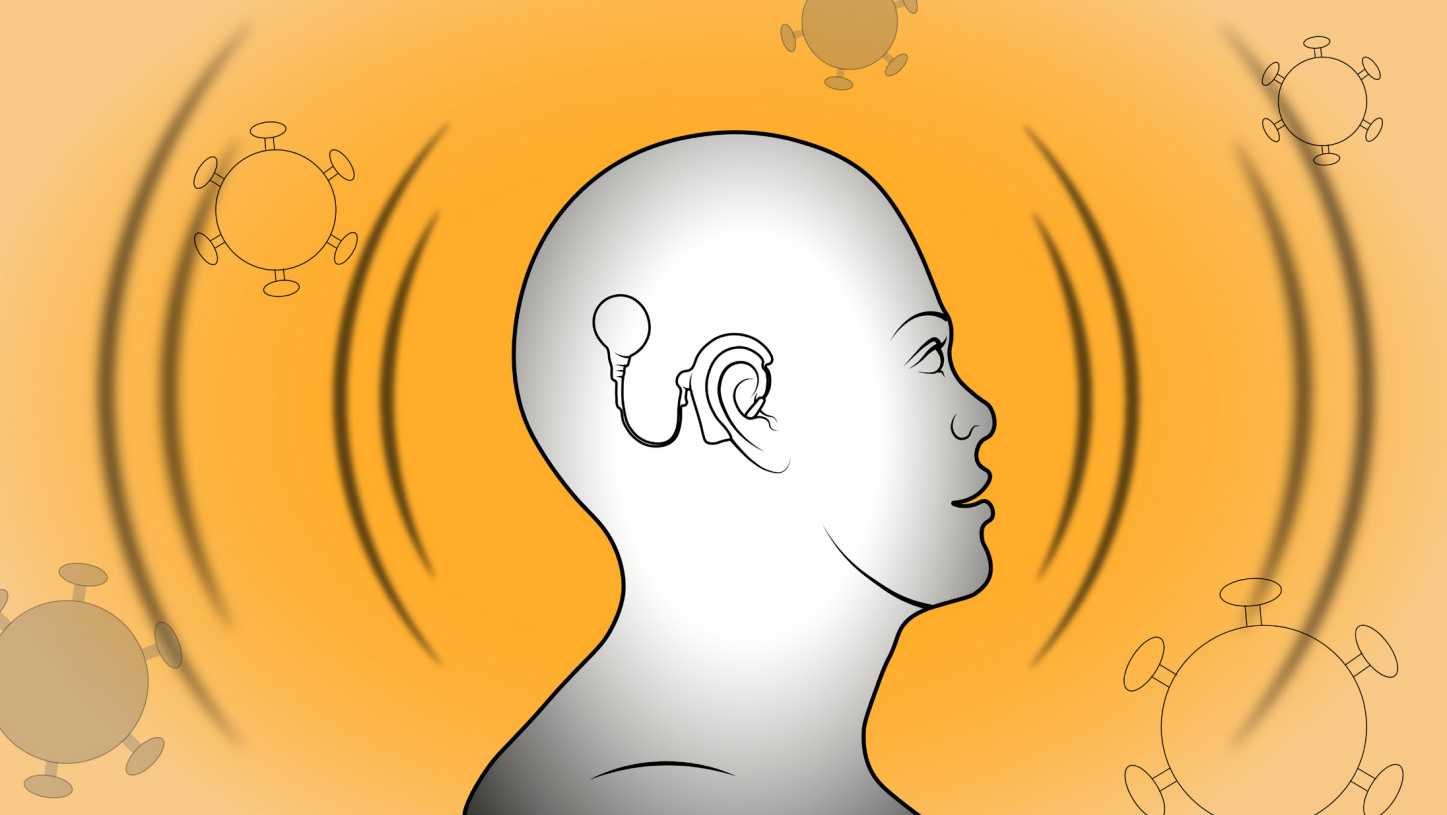
The pandemic exacerbated many challenges faced by scientists who are deaf or hard of hearing; it also ushered in some changes for the better.

Image credit: Monika Jasnauskaite (CC BY 4.0).

Ever since the COVID-19 outbreak had reached New York City in late February, I had been checking the newspapers every morning to get the latest number of cases and plot them. With the case rate doubling every 2.5 days, and vaccine production 12–18 months away at best, I knew that it was impossibly optimistic to expect normality to return within a few months. The world was going to be sheltering in place for the foreseeable future, masking, meeting remotely and social distancing. My ability to communicate with others was already tenuous. I felt as if it was about to be severed.

Born profoundly hearing impaired, I was fitted with hearing aids as a child and raised in the ‘oral world’. For many like me, hearing isn’t the problem; the problem is to make sense of the sounds. Picture yourself in a foreign country where you have only a rudimentary understanding of the language. Conversations, television programmes, what a service employee is trying to tell you… everything is incomprehensible except for a few words. If it’s quiet and you concentrate, you can partially piece things together. That’s my baseline; in hearing tests, my word recognition score is around 60%. In ideal situations, I can usually puzzle out what someone is saying by relying heavily on lipreading, context and non-verbal cues. If it’s noisy or these cues are not available, I understand nothing.

Barely a few days after the governor’s announcement, all my teaching and work meetings were taking place online. This quickly represented a challenge, as electronic or amplified speech sounds heavily distorted when re-amplified by my cochlear implant and hearing aid. Even when speakers had their webcam turned on and I could lipread, it was nearly impossible for me to understand what they were saying.

Real-time captioning, which would help solve my problems, was rarely supported by communications platforms at the time. To my dismay, I discovered that it was missing from the learning management system that my college used for teaching. Thankfully, I found an adequate substitute in Google Meet and I was able to continue communicating with my students. I had less success with some colleagues, who insisted on using their preferred platforms even though they lacked live captioning.

Online conferences and symposia were another challenge. When I alerted organizers that their software was not providing captions, most gamely tried to accommodate my needs but struggled to navigate technology issues. We once sat in an awkward, embarrassed silence for 20 minutes as the host of a Diversity, Equity, and Inclusion committee meeting attempted to find how to turn on captioning. I had been invited to talk about the challenges faced by deaf individuals; I think this real-time demonstration was possibly more effective than anything I could have planned to say. Not everybody was so cooperative, however. Some organizers did not respond to my requests or claimed that accommodations weren’t possible. One asked me to not “make life more complicated” for them — ironically, they were putting together a series of seminars about improving access to research for underrepresented minorities.

After a few of these incidents, I reached out to human resources for assistance. It took four months of persistent emailing before I was presented with a solution: I could schedule a live transcriptionist for meetings, as long as I provided notice 24 hours in advance. As it turned out, this ‘solution’ had several problems. Transcriptionists were slower and less accurate than automatic captioning, and only available during business hours. It was also a logistical challenge to get them into conferences, especially those with registration fees.

Fortunately, after a few months, most platforms started to incorporate live transcription into their software. With this, my whole world changed again. Meetings and conferences had always been difficult for me as I would struggle to locate the speaker, focus on their face, and figure out what they were saying before someone else chimed in. Online, it was easy to see everyone clearly and to follow the conversation with lipreading and captioning. With remote communication becoming mainstream, I can now participate in meetings and conferences to a greater extent than in the past.

These technological improvements don’t mean that I no longer rely on the kindness and cooperation of others. For example, I still reach out to conference organizers in advance to ensure that captioning will be provided, because it’s usually not turned on by default. Hearing loss is often an invisible disability, and hosts may not realise that attendees – including their colleagues – could have hearing difficulties. They may assume that people will request the accommodations they need, but many are embarrassed to ask or don’t want to ‘be a bother’. It’s better to provide these adjustments proactively and mindfully.

The pace of change can sometimes feel frustratingly slow, yet I often say that there has never been a better time to be deaf. The hearing aids I had as a child were primitive compared to the engineering marvels that carefully curate sounds for my ears today. When captioned media began its long, slow rollout in the 1990s, a new world opened for me, one where I could finally share my friends’ enthusiasm for the latest movies and dramas. Now the era of mainstreaming remote meetings has ushered in another sea change for me.

## Share your experiences

This article is a Sparks of Change column, where people around the world share moments that illustrate how research culture is or should be changing. Have an interesting story to tell? See what we’re looking for and the best ways to get in touch here.

